# A Composition Analysis and an Antibacterial Activity Mechanism Exploration of Essential Oil Obtained from *Artemisia giraldii* Pamp

**DOI:** 10.3390/molecules27217300

**Published:** 2022-10-27

**Authors:** Guiguo Huo, Xu Li, Mohamed Aamer Abubaker, Tingyu Liang, Ji Zhang, Xuelin Chen

**Affiliations:** 1 College of Life Science, Northwest Normal University, Lanzhou 730070, China; 2 Engineering Technology Research Center of Gansu Characteristic Plant Active Ingredient Products, Lanzhou 730070, China; 3 Institute of New Rural Development, Northwest Normal University, Lanzhou 730070, China; 4 Department of Biology, Faculty of Education, University of Khartoum, Khartoum 11111, Sudan

**Keywords:** *Artemisia giraldii* Pamp, essential oil, anti-oxidation, antibacterial activity

## Abstract

The goal of this work was to use the GC-MS technique to explore the chemical components of *Artemisia giraldii* Pamp essential oil (AgEo) and to uncover its antibacterial activity, specifically the antibacterial mechanism of this essential oil. There were a total of 63 chemical constituents in the AgEo, monoterpenes (10.2%) and sesquiterpenes (30.14%) were found to be the most common chemical components, with camphor (15.68%) coming in first, followed by germacrene D. (15.29%). AgEo displayed significant reducing power and good scavenging ability on hydroxyl radicals, 2,2-Diphenyl-1-picrylhydrazyl (DPPH) radicals, and 2,2′-Azinobis-(3-ethylbenzthiazoline-6-sulphonate (ABTS) radicals, according to antioxidant data. The diameter of the inhibition zone (DIZ) of AgEo against *S. aureus* and *E. coli* was (14.00 ± 1.00) mm and (16.33 ± 1.53) mm, respectively; the minimum inhibitory concentration (MIC) of AgEo against *E. coli* and *S. aureus* was 3 μL/mL and 6 μL/mL, respectively; and the minimum bactericidal concentration (MBC) of AgEo against *E. coli* and *S. aureus* was 6 μL/mL and 12 μL/mL, respectively. The antibacterial curve revealed that 0.5MIC of AgEo may delay bacterial growth while 2MIC of AgEo could totally suppress bacterial growth. The relative conductivity, alkaline phosphatase (AKP) activity, and protein concentration of the bacterial suspension were all higher after the AgEo treatment than in the control group, and increased as the essential oil concentration was raised. In addition, the cell membrane ruptured and atrophy occurred. The study discovered that AgEo is high in active chemicals and can be used as an antibacterial agent against *E. coli* and *S. aureus*, which is critical for AgEo’s future research and development.

## 1. Introduction

Recently, as people’s awareness of food safety and environmental protection has grown, various active compounds from natural plants have been isolated and used in a variety of applications, including antibacterial and fresh-keeping. Research has revealed that *Artemisia* extracts and essential oils have potent antibacterial properties [1,2,3].

*Artemisia giraldii* Pamp, a herbaceous plant, belonging to the Artemisia genus in the Compositae family, and has a distinct scent. Distributed in some areas in China, such as Mongolia [4], Hebei, Shanxi, Shaanxi, Ningxia, Gansu, and Sichuan (northwest) provinces [4,5]. It has widespread pharmacological activities, santolinylol; extract from *Artemisia giraldii* Pamp has been reported to have antifungal activity [6]. 4′,6,7-trihydroxy-3′,5′-dimethoxyflavone and 5′,5-dihydroxy-3′,4′,8-trimethoxyflavone were isolated from *Artemisia giraldii* and shown to have antibiotic activity against *Staphylococcus aureus, Sarcina lutea*, *Escherichia coli*, *Pseudomonas aeruginosa*, *Proteus* sp. *Aspergillus flavus*, and *Trichoderma viride* [7]. The essential oils derived from *Artemisia giraldii* has insecticidal activity against Sitophilus zeamais [8]. Xue Yang et al. isolated 10 sesquiterpenes from *Artemisia giraldii* var. longipedunculata [9], sesquiterpene lactones exhibit a wide range of biological activities, such as antitumor, anti-inflammatory, analgesic, antiulcer, antibacterial, antifungal, antiviral, antiparasitic, and insect deterrent [10].

However, there is yet to be any evidence of its essential oil antibacterial action. The chemical components of the essential oil from *Artemisia giraldii* Pamp were investigated further in this study, as well as its antimicrobial activity, in order to provide a reference and theoretical basis for the development and use of the essential oil from *Artemisia giraldii* Pamp, as well as for future research.

## 2. Results and Discussion

### 2.1. Chemical Compositions of the AgEo

**Figure 1:**
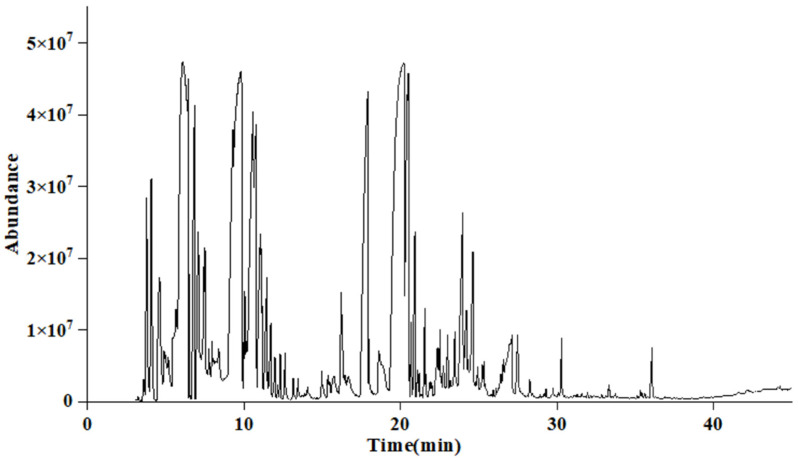
The total ion figure of AgEo.

The AgEo obtained by steam distillation was light white, without obvious color, with strong aroma, and the yield was 0.27% (w/w). In order to know the phytochemical contents, The chemical compositions of AgEo were identified by using GC-MS and the result was showed in Figure 1 and Table 1. There were 63 chemical constituents in the AgEo of which the monoterpenes (10.2%) and sesquiterpenes (30.14%) are the main components. Among all the chemical constituents, there are 21 chemical components were found to contain more than 1% among which Camphor (15.68%), Germacrene D (15.29%), Eucalyptol (14.18%), Terpinene-4-ol (7.57%) and Caryophyllene (6.40%) were the main characteristic constituents. This findings are similar to those of previous studies [8,11]. According to the study of Liang et al. [12], the main component of AgEo is 1,8-cineole (40.72%), camphor (22.50%), terpinen-4-ol (12.41%) and α-terpinol (4.14%). Chu et al. [8] showed that the main components of AgEo were β-pinene (13.18%), iso-elemicin (10.08%), Germacrene D (5.68%), and 4-terpineol (5.43%). Above two studies are similar to this study, but the types and contents of the main components in the essential oils in these three studies are different, which may be related to the different sampling sites and the locations from which the essential oils are extracted.

Up to now, no literature has reported antibacterial properties of AgEo, so this material cannot be directly compared with other materials. Studies have shown that *Lavandula latifolia* essential oil and camphor have good synergistic bacteriostatic effects on human pathogenic bacteria *S. aureus* and *L. monocytogenes* in vitro [12]. The chemical composition of essential oils of *Artemisia vulgaris* L., collected in Lithuanian has been reported. The results showed that germacrene D was revealed as the major constituent in the investigated oils and it had obvious biotoxicity to brine shrimp [13]. Previous studies have shown that the eucalyptol has a good inhibitory effect on *S. aureus* [14]. In addition, the main component of tea tree oil is terpinene-4-ol, which can affect the membrane function of *E. coli* and interfere with the metabolism of somatic cells. It can be seen from the above that camphor, germacrene D and eucalyptol have good biological activity, as the main component of AgEo, which may be related to its strong antibacterial activity.

### 2.2. Antioxidant Activity of the AgEo

#### 2.2.1. The Total Reducing Capacity

**Figure 2:**
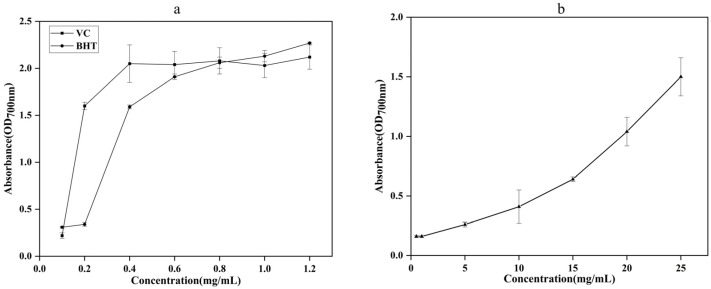
The total reducing capacity of VC, BHT (**a**) and AgEo (**b**).

The total lowering capacity of Vitamin C (VC), Butylated hydroxytoluene (BHT), and AgEo increased as their concentration rose, as shown in Figure 2. These findings suggested that the AgEo may stop free radicals from transferring electrons and transforming them into more stable compounds, effectively ending a chain of free radical processes. The total reducing capacity of VC and BHT was greater than that of the AgEo throughout the system, but once the AgEo reached a specific concentration, it showed a substantial reducing capacity as well, which was similar to the findings of Senol et al. [15].

#### 2.2.2. The Scavenging Ability of Hydroxyl Radical

**Figure 3:**
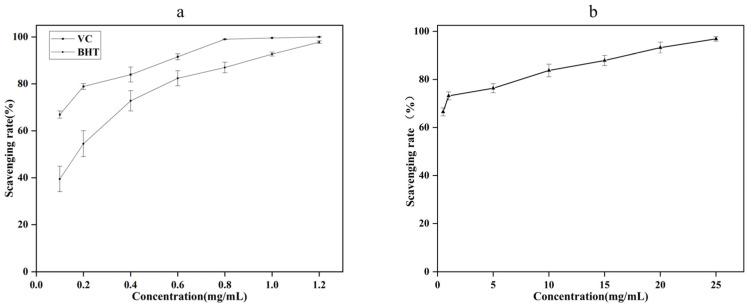
The clearance ability of VC, BHT (**a**) and AgEo (**b**) for hydroxyl radical.

As can be seen from Figure 3, when the concentration of AgEo is in the range of 0.5–25 mg/mL, its scavenging ability on hydroxyl radical increases with the increase of concentration. When the concentration of AgEo increased from 0.5 mg/mL to 25 mg/mL, the hydroxyl radical scavenging ability of AgEo increased from 66.48% to 96.87%. When the concentration of AgEo was 1 mg/mL, the clearance rate was 73.15%, when the concentration of VC and BHT was 1 mg/mL, the clearance rate was 99.60% and 92.70%, respectively. The results showed that AgEo had strong scavenging ability of hydroxyl radical.

#### 2.2.3. The Scavenging Ability of DPPH

**Figure 4:**
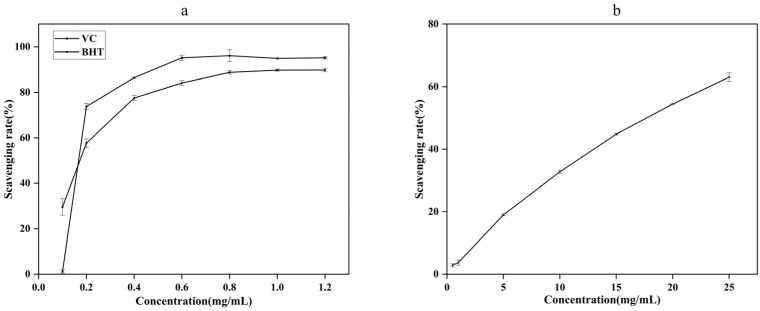
The clearance ability of VC, BHT (**a**) and AgEo (**b**) for DPPH radical.

As can be seen from Figure 4, when the concentration of VC and BHT ranged from 0.1 mg/mL to 0.6 mg/mL, the scavenging rate of VC and BHT on DPPH free radical increased rapidly with the increase of the concentration, when the concentration was greater than 0.6mg/mL, the scavenging ability was stable. The scavenging ability of AgEo on DPPH free radical had a dose-effect relationship with its mass concentration. When AgEo concentration increased from 0.5 mg/mL to 25 mg/mL, the scavenging rate of DPPH increased from 2.87% to 63.04%. When AgEo concentration was 1 mg/mL, DPPH clearance rate was 3.71%, when VC and BHT concentration was 1 mg/mL, DPPH clearance rate was 94.93% and 89.75%, respectively. The results showed that DPPH clearance rate (%) VC > BHT > AgEo. Low concentration of AgEo has A weak scavenging ability for DPPH, and when AgEo reaches A certain concentration, it has A strong scavenging ability for DPPH, which is similar to the results of Wei A [16].

#### 2.2.4. The Scavenging Ability of ABTS

**Figure 5:**
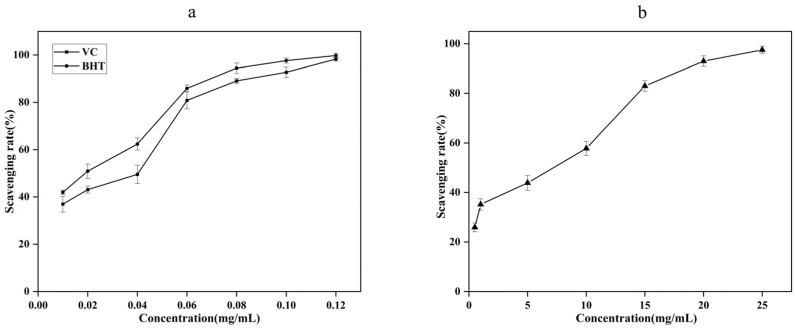
The clearance ability of VC, BHT (**a**) and AgEo (**b**) for ABTS radical.

As can be seen from Figure 5, the scavenging ability of VC, BHT and AgEo on ABTS was in a dose-effect relationship with its mass concentration. When the concentration of VC and BHT was 0.01 mg/mL, the ABTS clearance rates were 36.94% and 41.96%, respectively. When the concentration was 0.12 mg/mL, the ABTS clearance rates were 98.30% and 99.80%, respectively. AgEo concentration increased from 0.5 mg/mL to 25 mg/mL, and ABTS clearance rate increased from 25.94% to 97.55%. The results showed that the scavenging rate of VC and BHT on ABTS was stronger than that of AgEo, and AgEo had strong scavenging ability on ABTS free radical at a certain mass concentration.

### 2.3. The DIZ of the AgEo against Tested Strains

**Figure 6:**
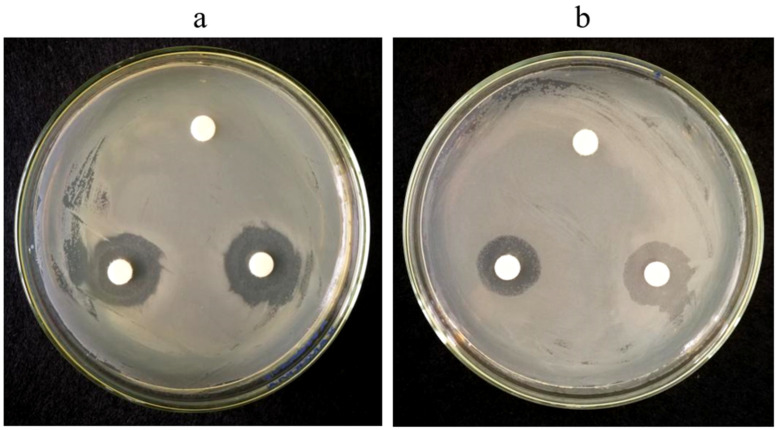
Antimicrobial effect of AgEo on *E. coli* (**a**) and *S. aureus* (**b**).

Antimicrobial effects of AgEo on the *E. coli* and *S. aureus* were given in Figure 6 and Table 2. Figure 6 shows that there is an obvious bacteriostatic zone around the filter paper added with AgEo, while the filter paper of the control group does not show bacteriostatic effect. The antibacterial zone of AgEo against *E. coli* and *S. aureus* was (16.33 ± 1.53) mm and (14.00 ± 1.00) mm, respectively. The antibacterial effect of AgEo against *E. coli* was stronger than that of AgEo against *S. aureus*.

### 2.4. MIC and MBC of the AgEo against Tested Strains

The MIC and MBC values of AgEo are shown in Table 3. AgEo exhibited the antibacterial activities against *E. coli* and *S. aureus*. In addition, negative control did not showed any antibacterial effect. The MIC values of AgEo against *E. coli* and *S. aureus* were 3 and 6 μL/mL while the MBC values were 6 and 12 μL/mL, respectively.

### 2.5. Growth Curves of the AgEo against Tested Strains

**Figure 7:**
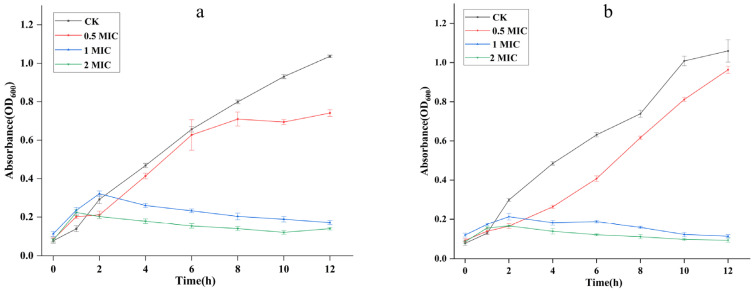
Effect of the AgEo on *E. coli* (**a**) and *S. aureus* (**b**) growth.

For further clarify the antibacterial activity of AgEo against *E. coli* and *S. aureus*, the time curves of them growth were plotted, as showed in Figure 7. Under the treatment of 0.5 MIC of AgEo, the inhibition effect of AgEo appears delayed, which may be mainly because of the low activity of bacteria in the early stage of growth, so the concentration of bacterial suspension is low and the inhibition effect is not significant. 2 MIC of AgEo can completely inhibited bacterial growth, these results demonstrated that antibacterial ability of AgEo obviously was increased with the dose.

### 2.6. Relative Electric Conductivity

**Figure 8:**
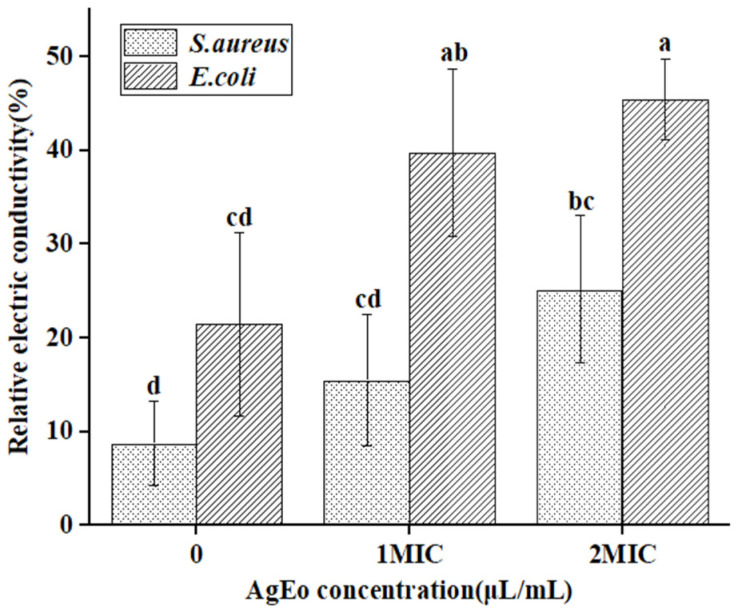
Effect of AgEo against cell membrane permeability of *E. coli* and *S. aureus.* Different normal letters indicate significant differences between treatments (*p* < 0.05, *n* = 3).

The plasma membrane of bacteria is the permeability barrier of bacteria, which plays a crucial role in regulating the concentration of sodium, potassium and calcium plasma inside and outside the cell, regulating cell energy metabolism and material transportation, and maintaining the stability of the intracellular environment [17]. In this study, *E. coli* and *S. aureus* were treated with AgEo at different concentrations, and their relative electrical conductivity was measured. The results are shown in Figure 8. The relative electric conductivity of *E. coli* and *S. aureus* treated with different concentrations of AgEo was higher than that of the control group, and the relative electric conductivity increased with the increase of AgEo concentration. Studies have showed that cinnamon essential oil can change the membrane permeability of *E. coli* and *S. aureus*, leading to a large amount of electrolyte leakage inside the cells, and to cell death [18]. In addition, fennel seed oil can also cause electrolyte leakage of dysentery bacillus, leading to cell death [19]. Therefore, in this study, the AgEo may also have changed the membrane permeability of the two bacteria, leading to a large amount of electrolyte leakage, thus showing an antibacterial effect.

### 2.7. The Leakage of Alkaline Phosphatase and Protein

**Figure 9:**
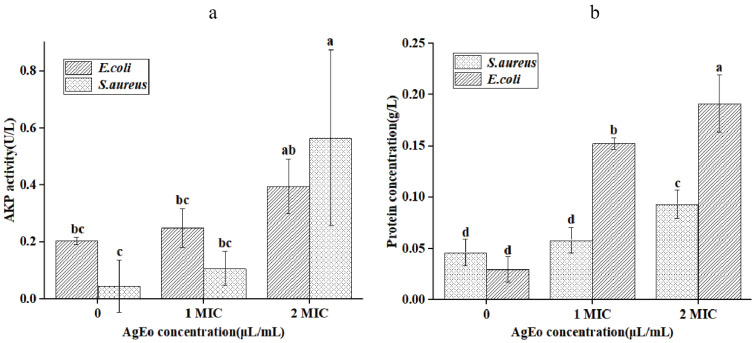
The alkaline phosphatase activity (**a**) and protein content (**b**). Different normal letters indicate significant differences between treatments (*p* < 0.05, *n* = 3).

Alkaline phosphatase and protein are significant biological macromolecules found in the membrane and cytoplasm of bacteria, and they play a crucial role in cell life [20]. In Figure 9, depicts the alkaline phosphatase activity and protein content in the supernatants of *S. aureus* and *E. coli* treated with AgEo at various doses. The alkaline phosphatase activity and protein content of two types of bacteria in the treatment group were higher than those in the control group, according to the findings. The alkaline phosphatase activity and protein content in the suspensions of the two types of bacteria increased in step with the increase in AgEo concentration, reaching their maximum value when AgEo concentration was 2 MIC. This shows that the essential oils may have disrupted the bacteria’s membrane structure, allowing active chemicals such as alkaline phosphatases and proteins to leak out of the bacteria’s cells, ultimately inhibiting the bacteria’s growth. The AgEo’s antibacterial effectiveness against *S. aureus* and *E. coli* was also validated in this study.

### 2.8. The Electron Scanning Micrograph

**Figure 10:**
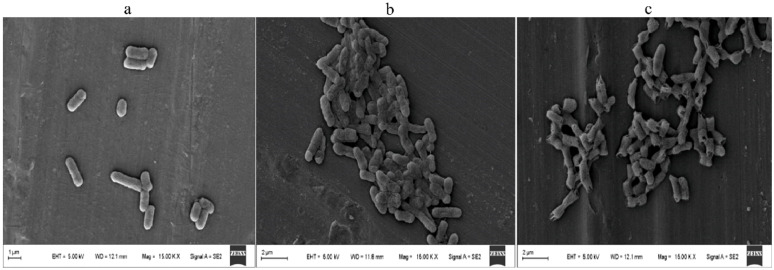
The electron scanning micrograph of *E. coli.* (**a**) The blank group after treatment for 4 h; (**b**) The MIC group after treatment for 4 h; (**c**) The MBC group after treatment for 4 h.

*E. coli* was used as the research subject and was treated with varied concentrations of AgEo using scanning electron microscopy in order to more intuitively perceive the harm of essential oil to bacterial structure (SEM). The *E. coli* in the blank group is rod-shaped, has a smooth surface, and has a relatively consistent morphology with no visible dents or breakages, according to Figure 10a,b is *E. coli* that has been treated with essential oil at the MIC concentration, and damage and atrophy dents have appeared on its surface. Figure 10c is *E. coli* that has been treated with MBC essential oil concentration. The cell’s surface is rough and uneven, and the cell membrane is clearly ruptured. The findings revealed that the AgEo might deform and destroy the *E. coli* cell membrane. As a result, the antibacterial action of AgEo could be linked to a change in cell membrane structure (Figure 10).

## 3. Materials and Methods

### 3.1. Plant Material and Bacterial Strains

The *Artemisia giraldii* Pamp was harvested from Zhenyuan County, Qingyang City, Gansu Province, China. It was identified by Dr Xuelin Chen (College of Life Sciences, Northwest Normal University). *S. aureus* and *E. coli* were provided by the Microbiology Laboratory of Northwest Normal University of China, and maintained in Luria-bertani (LB) agar slants at 4 °C. Two strains were cultured at 37 °C on nutrient agar (NA) or nutrient broth (NB) mediums.

### 3.2. Essential Oil Extraction

A total of (5 kg) dried *Artemisia giraldii* Pamp was ground into a powder form, distilled for 8 h using a steam distillation device and allowed the essential oil to produce completely. To eliminate imprints, the essential oil was extracted from the water and dried over anhydrous Na_2_SO_4_ before being filtered through 0.22 m filter membranes. Until used, the sterile essential oil was stored in firmly capped brown vials at 4 °C [21].

### 3.3. GC-MS Analysis

For the separation, a Hewlett Packard 5890 series II GC (Agilent Technologies, Santa Clara, CA, USA) was used, which was equipped with an HP-5 MS capillary column (300.25 mm, film thickness, 0.25 m) and an HP 5972 mass selective detector. With a mass scan range of m/z 30 to 550 at 70 eV, the mass selective detector was operated in electron-impact ionization (EI) mode. At a flow rate of 1 mL/min, helium was used as the carrier gas. The temperature was initially set at 70 °C, held for 1 min, then ramped at 3 °C/min to 180 °C, kept for 3 min, and finally increased at 5 °C/min to 230 °C, held for 5 min. Temperatures for the injector and MS transfer line were set at 230 and 250 degrees Celsius, respectively. A 1:10 split ratio was used to manually inject a sample of 1 mL of 1% essential oil.

### 3.4. Test of Antioxidant Ability

#### 3.4.1. The Test of Ferric Ion Reducing Antioxidant Power (FRAP)

Refer to relative Ardestani’s method [22], take 0.3 mL AgEo ethanol solution of different concentration gradient to join 1.5 mL 0.2 mol/L PBS (pH= 6.6), then add 1.5 mL potassium ferricyanide solution of 1%, shake well after 50 ℃ water bath pot incubation in 20 min, and ice water cooling rapidly. Finally to the mixture, in turn, add 1.5 mL trichloroacetic acid solution of 10%, 0.6 mL ferric chloride solution of 0.1% and 3 mL distilled water. After fully reaction 10 min, centrifuge 5 min under 3000 r/min. After let stand, using anhydrous ethanol as a blank zero, the absorbance at 700 nm was determined. VC and BHT were used as positive controls, and the higher absorbance under the same condition, the stronger the reducing ability was, the experiment was repeated for 3 times in each group.

#### 3.4.2. The Scavenging Ability of Hydroxyl Radical

Refer to the relative method [23], absorb 1 mL of AgEo ethanol solutions of different mass concentrations, then add 1 mL 9 mmol/L ferrous sulfate solution, 1 mL 8.8 mmol/L hydrogen peroxide and 1 mL 9 mmol/L salicylic acid solution successively. After fully mixed, the absorbance at 510 nm was measured as A_1_. The AgEo ethanol solution was replaced with equal volume of distilled water, and the absorbance at 510 nm was measured as A_0_. The above salicylic acid solution was replaced with an equal volume of distilled water, and the absorbance at 510 nm was measured as A_2_. VC and BHT were used as positive control, the experiment was repeated for 3 times in each group, and the hydroxyl radical clearance rate was calculated according to the formula below.

Hydroxyl radical scavenging rate (%) = [1 − (A_1_ − A_2_)/A_0_] × 100% (1)

#### 3.4.3. The Scavenging Ability of DPPH

According to the relative methodology [24], a specific amount of DPPH was weighed and made into a 0.1 mmol/L DPPH ethanol solution, which was then stored in a brown bottle for subsequent usage. After shaking and reacting for 20 min away from light, 2 mL of AgEo ethanol solutions of different mass concentration gradients were added to the same volume of DPPH ethanol solution in succession, and the absorbance at 517 nm was recorded, designated by A_1_. After shaking, a separate AgEo ethanol solution with a different mass concentration gradient was added to the same volume of ethanol solution, and its absorbance at 517 nm was measured, denoted by A_2_. After blending 2 mL DPPH ethanol solution with 2 mL ethanol, the absorbance at 517 nm was indicated as A_0_. Positive controls were VC and BHT, and the experiment was performed three times in each group, with the DPPH free radical clearance rate estimated using the formula below.

DPPH free radical scavenging rate (%) = [1 − (A_1_ − A_2_)/A_0_] × 100% (2)

#### 3.4.4. The Scavenging Ability of ABTS

According to methods [25], an equal volume of 7 mol/L ABTS solution was mixed with 2.45 mol/L potassium persulfate solution, and the reaction was carried out at room temperature for 15 h under dark conditions. After that, the mixture was diluted with ethanol until the absorbance at 734 nm was A_0_ [0.700 (0.002)]. After shaking and mixing for 20 min, the absorbance at 734 nm was measured, and the ABTS free radical clearance rate was calculated using the formula below.

ABTS free radical scavenging rate (%) = [(1 − A_1_)/A_0_] × 100% (3)

### 3.5. Determination of DIZ

The DIZ was determined by method of filter paper diffusion [26]. The AgEo was filtered through 0.22 mm Millipore filters (Qingfeng Filter Equipment Material Co., Ltd., Ji’an, China) 100 μL suspension of bacteria (1 × 10^7^ CFU/mL) cultured overnight was spread on the LB agar plate. Three filter paper (6 mm in diameter) were placed on the inoculated agar, two of them was added 10 μL essential oil by a micropipette and one was added with sterile water as control. The DIZ was measured after 24 h of in curation at 37 °C. Tests were performed in triplicate [19].

### 3.6. Determination of MIC and MBC

The MIC and MBC of AgEo was measured by bifold dilution method as recommended by the literature [27,28]. The AgEo was mixed with Tween 80 according to 5:1, and proper amount of culture solution was added. The AgEo was diluted step by step by double dilution method. Finally, the concentration of the volatile oil in the 96-well plate reached a series of concentration gradients, such as 12, 6, 3, 1.5 and 0.75 μL/mL, respectively. Microspores added only bacterial suspension and culture solution were used as positive control, and microspores added only Tween 80 and culture solution were used as negative control. Finally, the 96-well plate was placed in an incubator and cultured at 37 ℃ for 24 h. The minimum concentration of essential oil with no obvious bacterial growth was the minimum inhibitory concentration, and the minimum concentration of essential oil with no growth of bacteria after coating was the minimum bactericidal concentration [29].

### 3.7. Growth Curves

The growth curve assay method according to the method described by Zeng et al. [30], with slight modifications in briefly, logarithmic phase *S. aureus* and *E. coli* was diluted to 1 × 10^7^ CFU/mL with nutrient broth. The AgEo was dissolved in tween 80 and added to the cultures to keep the final concentrations of 0.5 MIC, 1 MIC and 2 MIC, only pathogens bacteria were added to the nutrient broth as a control. The cultures were incubated in nutrient broth at 37 ℃ and 120 rpm. At 0, 2, 4, 6, 8, 10 and 12 h, the absorbance of samples at 600 nm was measured.

### 3.8. Relative Electric Conductivity

Cell membrane permeability of *S. aureus* and *E. coli* were treated with different concentrations of essential oil, then determined according to Kongm et al. [31], *S. aureus* and *E. coli* were cultured in culture medium at 37 ℃ for 12 h, and then centrifuged at 4000 rp/min for 10 min. The electrical conductivity of 5% glucose solution heated in boiling water for 5 min was recorded as L0, and the electrical conductivity of different concentrations of essential oil (0, 1 MIC, 2 MIC) added into 5% glucose solution was recorded as L1. The cells were washed with 5% glucose solution for 3 times to make their conductivity close to that of 5% glucose solution, and then added with different concentrations of essential oils (0, 1 MIC, 2 MIC), completely mixed and cultured at 37 ℃ for 24 h, and the conductivity was recorded as L2. The relative conductivity was calculated by the following formula:

Relative electric conductivity (%) = [(L2 − L1)/L0] × 100%(4)

### 3.9. Determination of Alkaline Phosphatase, Protein and Nucleic Acid Content

The overnight cultured *S. aureus* and *E. coli* suspensions were diluted to (1 × 10^7^ CFU/mL), and an appropriate amount of the suspensions were added into the shaker tube and centrifuged at 5000 r/min for 5 min. After the supernatant was removed and washed twice with phosphate balanced solution (PBS), appropriate amount of PBS and AgEo was added into the shaker tube and make the concentrations of AgEo was 0, 1 MIC and 2 MIC, respectively. The sample was cultured at 37 ℃ and 120 r/min for 4 h, then centrifuged at 5000 r/min for 10 min, the contents of alkaline phosphatase, protein and nucleic acid in the supernatant were determined.

### 3.10. Scanning Electron Microscope (SEM)

To determine the efficacy of the essential oil and the morphological changes on the treated bacteria, the SEM observation was performed on the treated bacteria. The suspension of *E. coli* was prepared with a concentration of approximately 1 × 10^7^ CFU/mL, which was treated with essential oil with concentrations of 0, MIC and MBC at 37 ℃ for 4 h, and then centrifuged at 4 ℃ at 4000 rp/min for 10 min. The cells were washed with 0.1 M PBS (pH = 7.4) for 3 times. At 4 ℃, 2.5% (*v/v*) glutaraldehyde was fixed for 6 h. After washing with 0.1 M PBS for 3 times again, ethanol (30%, 50%, 70%, 80%, 90%, and 100%) was used for gradient dehydration for 15 min, followed by gold spraying treatment. Finally, morphology of the bacterial cells was observed on a scanning electronic microscope, 15 kV (JSM-7800F, JEOL, Tokyo, Japan) [17].

## 4. Statistical Analysis

All experiments were conducted in triplicates, averaged and presented followed by the standard deviation. Obtained results were statistically analyzed by SPSS software (version 22.0; IBM Corp., Armonk, NY, USA). One-way analysis of variance (ANOVA) and the Bonferroni statistical test were used to determine the significant differences at a significance level of *p* < 0.05.

## 5. Conclusions

1. There were a total of 63 chemical constituents in the AgEo of which the monoterpenes (10.2%) and sesquiterpenes (30.14%) were the main constituents. Among all chemical constituents, Camphor (15.68%), Germacrene D (15.29%), and Eucalyptol (14.18%) were the main characteristic constituents;
2. AgEo can effectively scavenge hydroxyl radicals, DPPH radicals and ABTS radicals, and has good antioxidant capacity;
3. AgEo is high in a range of active compounds with good inhibitory activity against *S. aureus* and *E. coli*. AgEo acts on the surface of bacteria, which can atrophy and rupture the bacterial cell membrane, leak intracellular biological macromolecules, such as alkaline phosphatase and protein, and disrupt the intracellular homeostasis, eventually leading to bacterial inactivation and death.

## Figures and Tables

**Table 1:** The chemical composition of AgEo.

NO	RT (min)	Compounds	Molecular Formula	IK	Relative Content (%)
1	3.786	α-Pinene	C_10_H_16_	939	1.41
2	4.085	Camphene	C_10_H_16_	954	1.46
3	4.594	β-Terpinene	C_10_H_16_	1049	1.56
4	4.927	6-Methyl-3,5-heptadiene-2-one	C_8_H_12_O	1074.9	1.19
5	6.101	Eucalyptol	C_10_H_18_O	1023	14.18
6	6.807	γ-Terpinene	C_10_H_16_	1057	3.84
7	7.472	1-methyl-4-(1-methylethylidene)-Cyclohexene	C_10_H_16_	1025	1.68
8	7.771	β-Terpineol	C_10_H_18_O	1127	0.49
9	7.988	Thujone	C_10_H_16_O	931	1.01
10	8.382	trans-1-methyl-4-(1-methylethyl)-2-Cyclohexen-1-ol	C_10_H_18_O	1123	0.72
11	9.781	Camphor	C_10_H_16_O	954	15.68
12	10.561	(-)-Terpinene-4-ol	C_10_H_18_O	1161	7.57
13	11.043	L-α-Terpineol	C_10_H_18_O	1189	2.24
14	11.423	2-Pentylcyclopentanone	C_10_H_18_O	1600	1.04
15	11.688	(-)-cis-Carvinol	C_15_H_26_O	-	0.53
16	11.966	Carveol	C_10_H_16_O	1188	0.30
17	12.313	D-Carvone	C_10_H_14_O	1244	0.20
18	12.632	2-isopropyl-5-methyl-3-Cyclohexen-1-one	C_10_H_16_O	1251	0.28
19	13.154	(-)-Perillaldehyde	C_10_H_14_O	1243	0.14
20	13.460	Benzyl acetate	C_9_H_10_O_2_	1141	0.14
21	14.071	*p*-Cymen-7-ol	C_10_H_14_O	1011	0.29
22	14.987	1,5,5-Trimethyl-6-methylene-cyclohexene	C_10_H_16_	1338	0.25
23	15.395	α-Borneol	C_10_H_18_O	-	0.26
24	15.727	3-Allyl-6-methoxyphenol	C_10_H_12_O_2_	1446	0.47
25	16.230	α-Copaene	C_15_H_24_	1397	1.19
26	16.691	Calarene	C_15_H_24_	1592	0.56
27	17.859	Caryophyllene	C_15_H_24_	1422	6.40
28	18.653	Humulene	C_15_H_24_	1456	1.32
29	20.154	Germacrene D	C_15_H_24_	1490	15.29
30	20.445	Bicyclogermacrene	C_15_H_24_	1496	4.04
31	20.900	β-Cadinene	C_15_H_24_	1491	1.26
32	21.559	1-allyl-2-methylene-Cycloheptanol	C_10_H_14_O	1491	0.55
33	21.912	Nerolidol	C_15_H_26_O	1548	0.24
34	23.018	8-propoxy-Cedrane	C_18_H_32_O	1652	0.59
35	23.473	octahydro-2,2,4,7a-tetramethyl-1,3a-Ethano(1H)inden-4-ol	C_15_H_24_O	1648	0.61
36	23.942	Cedrenol	C_15_H_24_O	1604	3.38
37	24.600	α-Cadinol	C_15_H_26_O	1589	1.35
38	24.940	Isoaromadendrene epoxide	C_15_H_24_O	1590	0.32
39	25.286	4-methylene-1-methyl-2-(2-methyl-1-propen-1-yl)-1-vinyl-Cycloheptane	C_15_H_24_O	-	0.58
40	27.071	1-(3-cyclopentylpropyl)-2,4-dimethyl-Benzene	C_15_H_24_	1188	2.28
41	27.478	Spathulenol	C_15_H_24_O	1619	0.66
42	28.266	1,5-diethenyl-3-methyl-2-methylene-(1.α.,3.α.,5.α.)-Cyclohexane	C_18_H_36_O	-	0.22
43	29.311	6,10,14-trimethyl-2-Pentadecanone	C_18_H_36_O	1842	0.14
44	29.759	5-Nonadecen-1-ol	C_19_H_38_O	1891	0.11
45	30.296	Sclareoloxide	C_18_H_30_O	1873	0.42
46	31.294	Hexadecanoic acid methyl ester	C_17_H_34_O_2_	1985	0.07
47	31.952	3,7,11,16-tetramethyl-Hexadeca-2,6,10,14-tetraen-1-ol	C_18_H_36_O	-	0.12
48	32.380	*n*-Hexadecanoic acid	C_16_H_32_O_2_	1942	0.10
49	32.862	8.α.,13-propylene oxide-14-ene	C_18_H_36_O	-	0.03
50	33.330	α-Curcumin	C_21_H_20_O_6_	1471	0.13
51	33.724	2,3,5,8-tetramethyl-1,5,9-Decatriene	C_14_H_24_	1485	0.08
52	35.346	1,2-Cyclohexanedicarboxylic acid di(3-methylphenyl) ester	C_22_H_24_O_4_	-	0.11
53	35.611	Methyl linolenate	C_19_H_32_O_2_	2077	0.04
54	36.059	Phytol	C_20_H_40_O	2104	0.36
55	36.894	2-heptadecyl-4,5-dihydro-1H-Imidazole	C_20_H_40_N_2_	1498	0.02
56	40.703	1-Methyl-6-(3-methylbuta-1,3-dienyl)-7-oxabicyclo [4.1.0]heptane	C_12_H_18_O	2647.8	0.03
57	41.626	Docosane	C_22_H_46_	2200	0.06
58	42.169	2-Hydroxy-2,4,4-trimethyl-3-(3-methylbuta-1,3-dienyl)cyclohexanone	C_14_H_22_O	-	0.09
59	43.221	15,17-Dotriacontadiyne	C_32_H_58_	3200	0.13
60	43.737	Alloaromadendrene	C_15_H_24_	1490	0.08
61	44.233	2-Dodecen-1-yl(-)succinic anhydride	C_16_H_26_O_3_	1966	0.07
62	44.647	2,2-dimethyl-,(3.β.,5.α.)-Cholest-7-en-3-ol	C_15_H_26_O_2_	3170	0.02
63	44.959	Caparratriene	C_15_H_26_	1493	0.02
Total					100.00

**Table 2:** Antimicrobial effects of AgEo on *E. coli* and *S. aureus* (mm diameter zone).

Strains	Diameter of Inhibition Zones (mm) ^a^	CK (mm)
*E. coli*	16.33 ± 1.53 a	0
*S. aureus*	14.00 ± 1.00 b	0

^a^ Indicated as an average of triplicates ± standard error. Different letters (the same column) represent statistically significant differences between the means (*p* < 0.05).

**Table 3:** The MIC and MBC of the AgEo on *E. coli* and *S. aureus.*

Strains	MIC ^a^ (μL/mL)	MBC ^b^ (μL/mL)
*E. coli*	3	6
*S. aureus*	6	12

^a^ MIC, minimum inhibition concentration. ^b^ MBC, minimum bactericide concentration.

## Data Availability

The data presented in this study are available in article.

## Sample Availability

Samples of the compounds are available from the authors.

## Abbreviations

AgEo	Artemisia giraldii Pamp essential oil
DPPH	2,2-Diphenyl-1-picrylhydrazyl
ABTS	2,2′-Azinobis-(3-ethylbenzthiazoline-6-sulphonate
DIZ	diameter of the inhibition zone
MIC	minimum inhibitory concentration
MBC	minimum bactericidal concentration
AKP	Alkaline phosphatase
LB	Luria-bertani
NA	Nutrient agar
NB	nutrient broth
FRAP	Ferric ion reducing antioxidant power
DPPH	1,1-diphenyl-2-picryl-hydrazyl radical
ABTS	2,2′-Azinobis-(3-ethylbenzthiazoline-6-sulphonate
SEM	Scanning electron microscope
VC	Vitamin C
BHT	Bbutylated hydroxytoluene
